# Engineering hydrogel nanoparticles to enhance transdermal local anaesthetic delivery in human eyelid skin

**DOI:** 10.1039/c9ra06712d

**Published:** 2020-01-23

**Authors:** Bengi Ozkahraman, Krisztina Emeriewen, George M. Saleh, Nguyen Thi Kim Thanh

**Affiliations:** Hitit University, Faculty of Engineering, Polymer Engineering Department 19030 Corum Turkey; Moorfields Eye Hospital NHS Foundation Trust London EC1V 2PD UK; The National Insitute for Health Research Biomedical Research Centre at Moorfields Eye Hospital, UCL Institute of Opthalmology 11-43 Bath St London EC1V 9EL UK; Biophysics Group, Department of Physics and Astronomy, University College London Gower Street London WC1E 6BT UK ntk.thanh@ucl.ac.uk; UCL Healthcare Biomagnetic and Nanomaterials Laboratories, University College London 21 Albemarle Street London W1S 4BS UK

## Abstract

Herein, we focused on developing the feasibility of nano-enabled local anaesthetic (LA) delivery to anaesthetise the full thickness of eyelid skin. For this purpose a temperature-responsive hydrogel poly(*N*-vinylcaprolactam-*co*-hyaluronic acid) (p(VCL-*co*-HA)) was prepared through aqueous emulsion polymerization with a Food and Drug Administration (FDA) approved p(VCL) and hyaluronic acid (HA) showing remarkably high LA drug loading capacity.

## Abstract 2

Nano-enabled local anaesthetic delivery was achieved using FDA-approved materials with remarkably high loading capacity.

## Graphical abstract

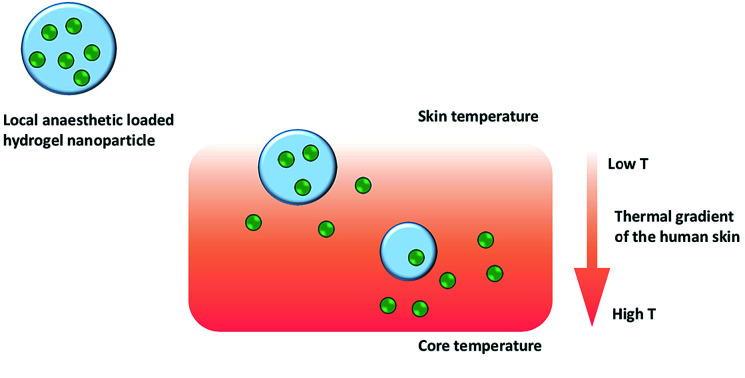

Eyelid surgery is most commonly performed under local anesthesia all over the world. Unfortunately not all patients experience local anesthetic injections in the same way. Many people have needle phobias and feel highly anxious at the prospect of an injection along with the pain associated with it, which adds to the overall trauma. Furthermore during prolonged procedures as the anesthetic effect wears off the pain returns, which adds to the traumatic experience. The aforementioned discomfort not only discourages the needle phobic patients but also other patients who have a low threshold for pain or are undergoing their first surgical procedure in the ophthalmic department. This fear may cause delays in seeking ophthalmic care early for serious conditions such as eyelid cancer and may also increase the amount of local anesthesia required for minor procedures which can have risks of systemic side effects from the anesthetic drugs. Both examples have significant impacts on the economy of national health care.

The current method of achieving sufficient dermal anesthesia in the eyelid for surgical procedures is subcutaneous injection of the LA drug lidocaine. An interest in non-invasive (needleless) LA drug delivery is well sought to minimize patient discomfort as well as surgical challenges such as intra-operative tissue distortion associated with the infiltration of anesthesia.^1,2^

The use of topical anesthetic products as an alternative for dermatological procedures on skin elsewhere in the body is well established.^3^ The most commonly used LA drug in the commercially available topical preparations is lidocaine which can be found for example in the form of a liposomal preparation of 4% lidocaine (LMX4 cream, Ferndale Laboratories Inc., Ferndale, MI, USA) or as an eutectic mixture of 2.5% lidocaine combined with 2.5% prilocaine (EMLA®, AstraZeneca AB, Södertälje, Sweden).^3^ Other products may contain tetracaine such as the 4% gel preparation Ametop or betacaine (Betacaine Enhanced Gel 4, Tiberius Inc, Tampa, FL).^4^

Though most of these preparations require a long application time and the need for occlusion to enhance deep penetration of the LA drug, the achieved anaesthesia is still insufficient to carry out procedures involving full thickness of eyelid skin.^3^ Furthermore eyelid dynamics prohibit the use of the aforementioned formulations that may cause ocular surface irritation or chemical injury in severe cases.^5^

In recent years, much interest has been given to nanocarriers that show potential for enhanced transdermal anaesthetic delivery *via* a range of routes (including the intracellular, intercellular and transappendageal) which is due to their small size. The carriers offer several advantages such as increased drug-loading capacity, entrapment efficiency and cumulative drug release.^6^

Hydrophilic polymeric networks that are capable of imbibing huge volumes of water and undergoing swelling and shrinkage to suitably facilitate controlled drug-release are called hydrogels. Nanogels are synthesized by the cross-linking of *N*,*N*′-methylenebisacrylamide and ammonium persulfate (APS) as an initiator using a radical polymerization technique. Their porosity and compatibility with aqueous environments make them highly attractive bio-compatible drug delivery vehicles. Hydrogels that are responsive to specific molecules, such as glucose or antigens, can be used as biosensors as well as drug delivery systems. New synthetic methods have been used to prepare homo- and co-polymeric hydrogels for a wide range of drugs, peptides, and protein delivery applications. HA is an FDA approved co-polymer and is an important component of the cellular matrix and various tissues that make up the organisms which have high moisture retention and high viscoelasticity. HA is widely used in anticancer drug delivery, since it is biocompatible, biodegradable, non-toxic, and non-immunogenic, it can be chemically modified to become a good drug carrier.^7,8^

For biomedical applications, poly(*N*-vinylcaprolactam)-based (p(VCL)) hydrogel nanoparticles are ideal as they have similar water content to natural tissue. In addition they are one of the most popular thermoresponsive polymers used in the cosmetic industry as their phase transition in response to temperature can be utilized to optimise skin application. The polymers are often synthetized as co-polymers with other chemicals to achieve additional benefits such as biotechnological applications due to their tunable size from nanometres to micrometres, a large surface area for multivalent bioconjugation and an internal network useful for incorporation of biomolecules or drugs.^9,10^

In this work we aim to explore the feasibility of nano-enabled LA delivery to anaesthetise the full thickness of the eyelid skin. For this purpose a temperature-responsive hydrogel poly(*N*-vinylcaprolactam-*co*-hyaluronic acid) (p(VCL-*co*-HA)) was prepared through aqueous emulsion polymerization with p(VCL) and HA for high LA drug loading capacity.

## Material and methods

### Materials

*N*-Vinylcaprolactam (VCL, 99%), sodium dodecyl sulfate (SDS), *N*,*N*′ methylenebis(acrylamide) (NMBA) and lidocaine HCl were obtained from Sigma-Aldrich, UK. HA and APS were purchased from Acrös Organics, UK. Visking® dialysis tubing with molecular weight cut-off (MWCO): 12–14 kDa and thickness: 2 mm was supplied from Medicell Membranes (London, UK). Ultrapure de-ionized water was used in all experiments.

### Synthesis of nanogels (NGs)

The p(VCL-*co*-HA) NGs were prepared *via* emulsion polymerization. In the first step, VCL, SDS and NMBA were cross-linked in water in a two-necked round flask with a magnetic stir bar and purged with nitrogen for 30 min. To start the polymerization APS was added into the flask. The mixture was stirred for 5 min before HA (in 25 mL water) was added into the reaction vessel and the polymerization was complete in a few hours. Dialysis with water was performed over 2 week to remove the excessive monomers. For the dialysis process, a cellulose membrane with a MWCO of 12–14 KDa was used and the water was changed twice daily. Three separate nanogel samples were synthetized with the amounts of NMBA, SDS and APS kept constant (Table 1).

**Table 1:** The amounts of reagents used for the syntheses of NGs

Nanogel code	VCL (g)	HA (g)	NMBA (g)	SDS (g)	APS (mg)
NG-1	1.96	0.04	0.06	0.02	46
NG-2	1.94	0.06	0.06	0.02	46
NG-3	1.92	0.08	0.06	0.02	46

After the dialysis the p(VCL-*co*-HA) polymeric particles were freeze-dried with the drug loading carried out by swelling 0.10 g of the polymers in 10 mL lidocaine HCL solution.

### Characterization of the NGs

#### Particle size, size distribution and zeta potential

The NGs were prepared at a concentration of 3 mg mL^−1^ at pH 5.5 and the particle size and size distribution of the p(VCL-*co*-HA) polymers were measured by dynamic light scattering (DLS) at both 25 °C and 37 °C to further determine their swelling behaviour. The surface zeta potentials of the polymeric particles were also attained by using a Zetasizer Nano ZS (Malvern Zetasizer ZS, UK) at both 25 °C and 37 °C.

#### Transmission electron microscopy (TEM)

The morphology and diameter of the p(VCL-*co*-HA) polymers were determined using a TEM 2500–12 000×, 120 kV (JEM 1200-EX, JEOL Ltd., JPN). The NG suspension was cast on a carbon coated copper grid and stained with a solution of 2% w/v phosphotungstic acid. The copper coated grid was dried at room temperature overnight before being placed into the TEM.

#### Lidocaine loading capacity

The drug loading capacity of the p(VCL-*co*-HA) polymers were measured with a Spectramax M2/M2e UV/Vis/NIR spectrophotometer (*λ* = 263 nm). After the polymers were loaded with lidocaine the supernatant layer was isolated and the concentration was determined by UV-Vis absorption spectroscopy. Prior to the analysis of the specimens calibration curves were prepared. The equation below was used to calculate the drug loading. The particle size, size distribution and zeta potential of the lidocaine loaded p(VCL-*co*-HA) polymers were also measured by DLS and TEM.
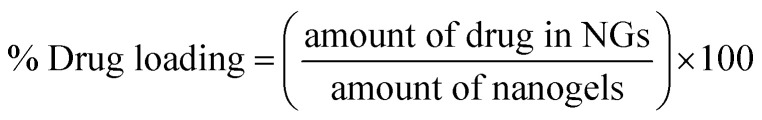


#### Fourier transform infrared (FTIR) spectroscopy studies

The spectroscopic analysis of the monomers (VCL and HA) and polymers (p(VCL-*co*-HA)) were carried out by Attenuated Total Reflectance-Fourier Transform Infrared (ATR-FTIR) spectrometer (PerkinElmer, USA). Further analysis with FTIR was carried out on the lidocaine-loaded p(VCL-*co*-HA) NGs.

#### Lower critical solution temperature (LCST) behaviours of NGs

The phase transition properties of the p(VCL-*co*-HA) NGs were assessed at 25 °C and 37 °C using the UV-Vis spectrophotometer at a wavelength *λ* = 452 nm.

## Results and discussion

### Particle size, size distribution and zeta potential

**Fig. 1:**
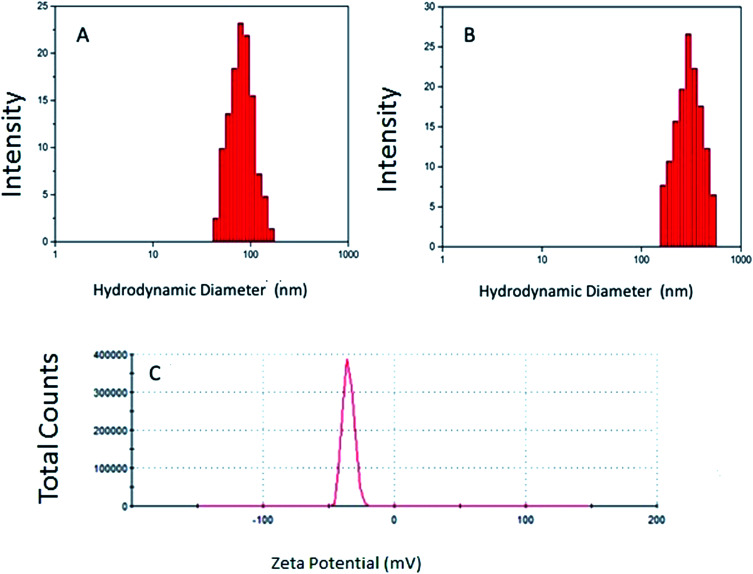
The particle size of the NG-1 measured by DLS at 25 °C (A) and 37 °C (B) with DLS, the zeta potential measurements obtained at pH 5.5 with a zeta sizer (C).

Fig. 1 shows the hydrodynamic diameters of the NG1 at 25 °C and 37 °C, and its zeta potential at pH = 5.5. The data of all three NG formulations including the particle size determined by TEM are summarised in Table 2. Analysis of the DLS measurements shows an increase in the particle size and size distribution as the p(VCL) content decreased and the HA content increased. The smallest particle diameter and narrowest size distribution was measured with NG-1 (128.0 ± 0.1 nm) and the largest particle diameter and widest size distribution is seen with NG-3 (205.0 ± 0.1 nm).

**Table 2:** The particle size and size distribution of the NGs measured at 25 °C and 37 °C with DLS, the zeta potential measurements obtained at pH 5.5 and the measured NG diameters with TEM at 2500× magnification

Polymer code	*d*_DLS_ (nm) 25 °C	PDI	*ζ* (mV) at pH 5.5	*d*_DLS_ (nm) at 37 °C	PDI	*d*_TEM_ (nm)
NG-1	128	0.097	−16.9	246	0.138	120
NG-2	189	0.104	−20.8	290	0.154	190
NG-3	205	0.121	−35.1	352	0.168	205

The stability of the formulations was estimated by measuring the zeta potentials of the NGs at pH 5.5. All three samples yielded negative potentials with the lowest value gained from the NG-3 (−35.1 mV) and the highest from the NG-1 (−16.9 mV) formulation. The measured zeta potential values indicate that NG-3 is the most stable of the three NGs synthesised. The negative surface charge is due to the presence of the sulphate groups from the APS and the polymerized carboxylic acid groups contained within the HA.^11,12^

During the experiments the APS component of each NG was kept constant whilst the HA amount was changed. It was observed that the zeta potential of the nanoparticles decreased as the HA content was increased (Tables 1 and 2). Thus, by increasing the number of carboxyl groups on the surface of the polymeric NGs, we were able to increase the negative charge of the particles and through this the stability of the molecule was also increased.

The data displayed in Table 2 shows that by decreasing the temperature of the nanogel formulations, the diameter of the p(VCL-*co*-HA) NGs decreased, for example for NG-1, it shrinks from 246 nm at 37 °C to 128 nm at 25 °C. The decrease in diameter is due to a change in the hydrodynamic balance occurring between the hydrophobic and hydrophilic components. As reported in literature, during phase transition in polymeric particles an increase in hydrophobic components can be seen due to the destruction of the hydrogen bonds.^8^ The increase in hydrophobic content can reduce the mobility of the hydrophilic chains and thus ensures a more sterically stable NG.

### Transmission electron microscopy (TEM)

**Fig. 2:**
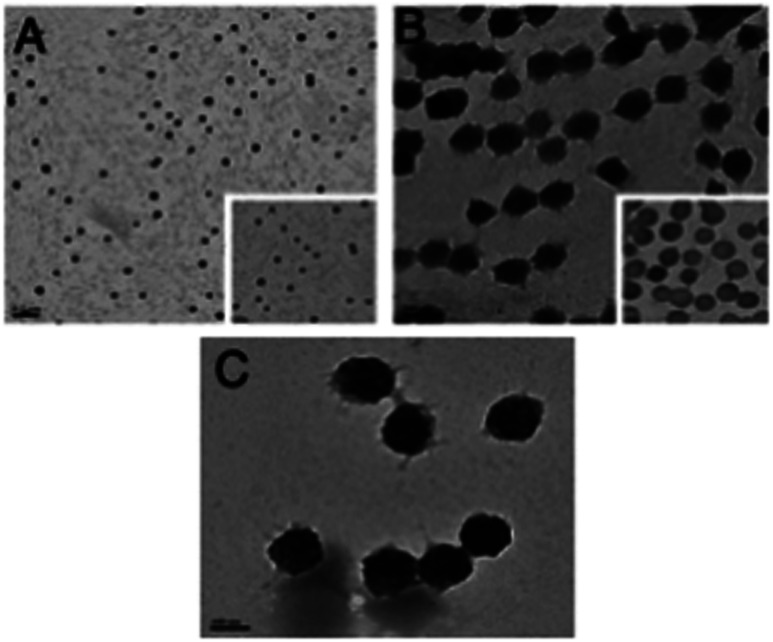
TEM images of the three nanogel formulations (A) NG-1, (B) NG-2 and (C) NG-3, scale bar = 100 nm.

The size of the p(VCL-*co*-HA) polymeric particles in the NGs obtained by TEM in Fig. 2 corroborates the trend of sizes determined by DLS. The number of acid groups (HA) incorporated into the NGs correlates with the measured NG diameter.

It is worth mentioning that the apparent diameter of each NG measured by TEM is smaller than that obtained by DLS and this is due to the shrinkage of the NGs in the drying process used for imaging.

Fig. 2 shows all three NGs have a spheroidal morphology. The measured particle size of NG-1, NG-2 and NG-3 can be seen in Table 2, which are 128 nm, 189 nm and 205 nm, respectively.

### FTIR studies

**Fig. 3:**
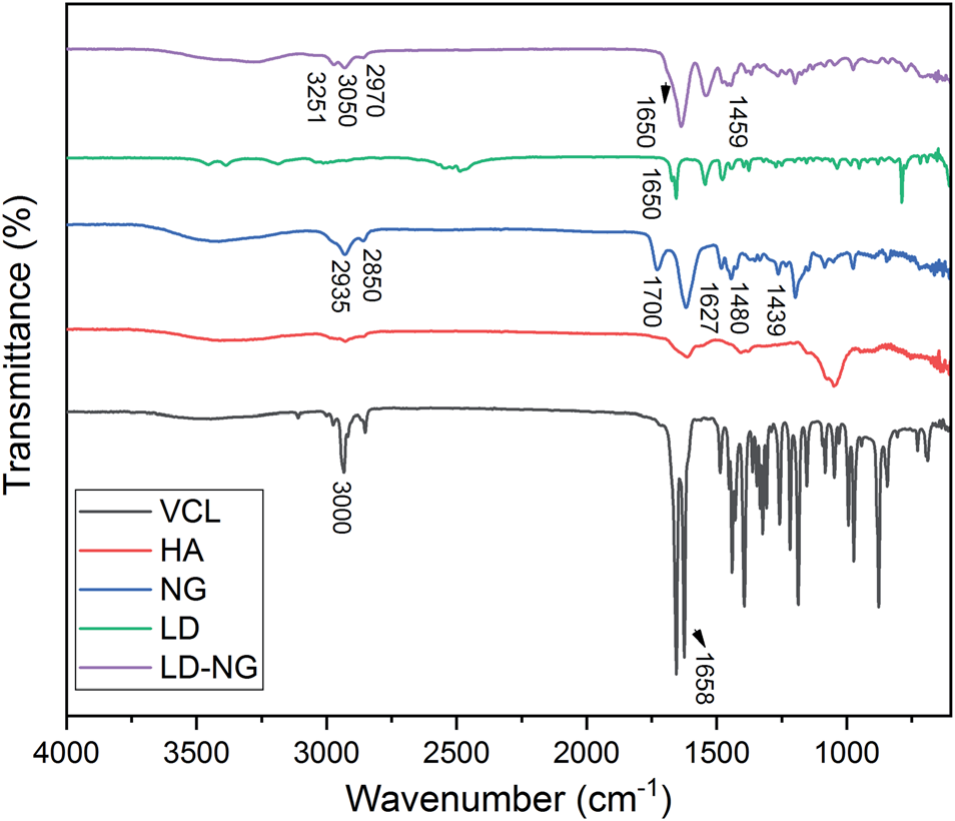
The FTIR spectra of VCL, HA, NG, LD and lidocaine loaded nanogel (NG-LD).

The chemical structures of the polymeric p(VCL-*co*-HA) NGs were characterized by FTIR spectroscopy by identifying characteristic peaks (Fig. 3). For NGs, the peaks at 2935, 2850 and 1439 cm^−1^ are due to the C–H groups. A characteristic peak identifying the presence of an amide is present at 1627 cm^−1^ with another peak at 1480 cm^−1^ due to the C–N present within the structure of the p(VCL-*co*-HA) NGs.^13^ Confirmation of the successful incorporation of the HA can be seen by the peak at 1700 cm^−1^ (COOH) of the p(VCL-*co*-HA) NGs.^14^

Additional conformation of the successful synthesis of the NGs can be seen in the lack of peaks at 1658 cm^−1^ (C=C) and 3000–3100 cm^−1^ (C=H and CH_2_) which are present in the VCL monomer (Fig. 3).^14,15^

Incorporation of the lidocaine in NGs is shown in Fig. 3 (top line) with a strong peak at 1650 cm^−1^ due to the amide group which was also seen in the pure lidocaine. In addition to the amide group peak, there are also characteristic peaks at 3251 cm^−1^ (H–N–C=O), 3050 and 2970 cm^−1^ (aromatic C–H), 2970 cm^−1^ (aliphatic C–H) and finally at 1650 and 1495 cm^−1^ (C=O) which proves that lidocaine has been loaded into the NGs.^16^

### Lower critical solution temperature (LCST) behaviours of NGs

**Fig. 4:**
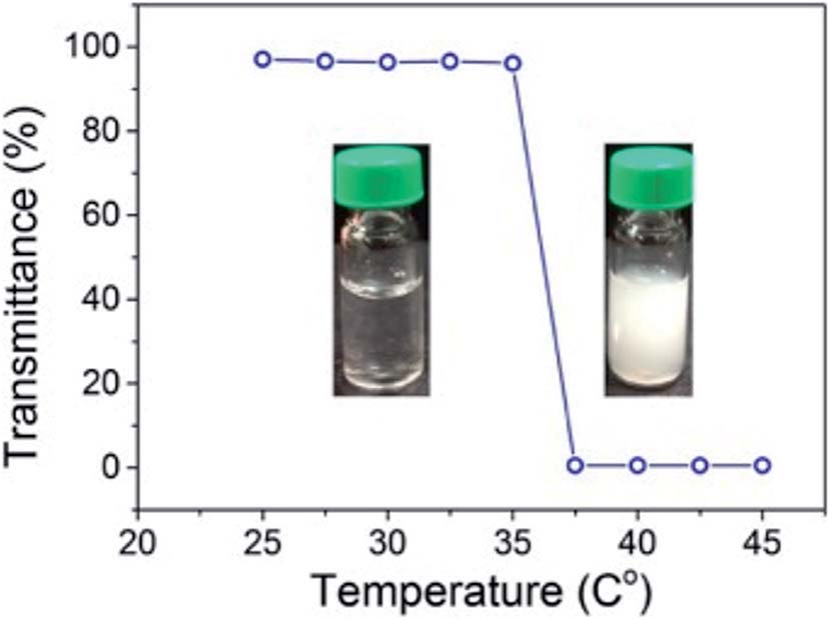
The transmittance of the lidocaine-loaded nanogel formulations (NG1-3) and photographs of a NG at 25 °C and 37 °C.

The LCST behaviour of the NGs were studied at 1.0% concentration. The turbidimetric method (cloud point measurement) was used to determine the LCST of the NGs. The cloud points of the NGs were determined using transmittance (%) *versus* temperature plots. During the experiments it was found that at temperatures of 35–37 °C the solution turns cloudy (Fig. 4).

PVCL is a thermo-responsive and biocompatible polymer that has a reversible volume phase transition temperature (VPTT) of 32–34 °C. In this study, we synthesized VCL-based copolymeric and terpolymeric nanogel. The aforementioned volume-phase transition temperature at which the nanogel particles collapse, depends on the solvent–polymer (hydrophilic) interactions and polymer–polymer (hydrophobic) interactions. The hydrophilicity of the polymer chain increased when the HA content increased; thus, the balance of attractive and repulsive forces is changed at a higher temperature, resulting in a higher LCST.

The *N*-isopropylacrylamide (NIPAM) contents in NGs also have a key influence on their phase transition behaviours. It can be seen in Fig. 4 that the phase transition curves become sharper as the poly(*N*-isopropylacrylamide) (PNIPAM) content increased, this effect was visible at all pH ranges investigated.

Based on these results, we can say that LCST values of NGs are in the range of the physiological temperature (35–37 °C) of the human body, which is an advantage for their application on the human skin.

## Conclusions

In this study, thermo sensitive nanogels were synthesized and characterized for local anesthetic (LA) drugs. Due to the presence of PVCL, nanogels showed the LCST at 35–37 °C. By FTIR we have confirmed that the lidocaine HCl is successfully loaded into the nanogel. The size of the most stable nanogel (NG-3) was found having size of 205 nm and 352 nm, at 25 °C and 37 °C respectively. TEM analysis confirmed that nanogels were spherical in shape. This in turn could be useful for use in eyelid and other topical surgeries where the nanogel formulations have shown potential in transdermal anesthetic delivery.

## Disclaimer

The views expressed are those of the author(s) and not necessarily those of the NHS, the NIHR or the Department of Health.

## Conflicts of interest

There are no conflicts to declare.

## Supplementary Material
